# Clinical, FDG and amyloid PET imaging in posterior cortical atrophy

**DOI:** 10.1007/s00415-015-7732-5

**Published:** 2015-04-11

**Authors:** Tarun D. Singh, Keith A. Josephs, Mary M. Machulda, Daniel A. Drubach, Liana G. Apostolova, Val J. Lowe, Jennifer L. Whitwell

**Affiliations:** Department of Neurology, Mayo Clinic, 200 First St. SW, Rochester, MN USA; Department of Neuropsychiatry (Neuropsychology), Mayo Clinic, Rochester, MN USA; Department of Radiology, Mayo Clinic, Rochester, MN USA; Department of Neurology, University of California Los Angeles, Los Angeles, CA USA

**Keywords:** PCA, FDG-PET, Cerebral hypometabolism, Clinical findings, Early PCA

## Abstract

The purpose of this study was to identify the clinical, [^18^F]-fluorodeoxyglucose positron emission tomography (FDG-PET) and amyloid-PET findings in a large cohort of posterior cortical atrophy (PCA) patients, to examine the neural correlates of the classic features of PCA, and to better understand the features associated with early PCA. We prospectively recruited 25 patients who presented to the Mayo Clinic between March 2013 and August 2014 and met diagnostic criteria for PCA. All patients underwent a standardized set of tests and amyloid imaging with [^11^C] Pittsburg compound B (PiB). Seventeen (68 %) underwent FDG-PET scanning. We divided the cohort at the median disease duration of 4 years in order to assess clinical and FDG-PET correlates of early PCA (*n* = 13). The most common clinical features were simultanagnosia (92 %), dysgraphia (68 %), poly-mini-myoclonus (64 %) and oculomotor apraxia (56.5 %). On FDG-PET, hypometabolism was observed bilaterally in the lateral and medial parietal and occipital lobes. Simultanagnosia was associated with hypometabolism in the right occipital lobe and posterior cingulum, optic ataxia with hypometabolism in left occipital lobe, and oculomotor apraxia with hypometabolism in the left parietal lobe and posterior cingulate gyrus. All 25 PCA patients were amyloid positive. Simultanagnosia was the only feature present in 85 % of early PCA patients. The syndrome of PCA is associated with posterior hemisphere hypometabolism and with amyloid deposition. Many of the classic features of PCA show associated focal, but not widespread, areas of involvement of these posterior hemispheric regions. Simultanagnosia appears to be the most common and hence sensitive feature of early PCA.

## Introduction

Posterior cortical atrophy (PCA) is a neurodegenerative disorder in which the onset of dementia is characterized by the development of higher order visual deficits [1]. This disease is often accompanied by visuospatial and visuoperceptual impairments, alexia, features of Bálint’s syndrome, Gerstmann’s syndrome and transcortical sensory aphasia, whereas episodic memory is relatively preserved or only mildly impaired [2]. These clinical features differ from those of typical dementia of the Alzheimer’s type (DAT) as they show preservation of memory, insight, and judgment until late in the clinical course, when the clinical features of PCA and DAT overlap [3]. The most frequent pathological findings in PCA are tau neurofibrillary tangles and beta-amyloid neuritic plaques which are characteristic of Alzheimer’s disease [4], although other pathologies have been described in PCA including corticobasal degeneration, diffuse Lewy body disease, and Creutzfeldt-Jakob disease [5–7]; frontotemporal dementia with progranulin mutation has also been reported [8].

The use of functional neuroimaging, using [^18^F]-fluorodeoxyglucose positron emission tomography (FDG-PET) allows the in vivo assessment of brain metabolism. Previous FDG-PET studies in PCA patients have shown prominent hypometabolism in the occipito-parietal region with variable involvement of the frontal eye fields and the relative sparing of the frontal and medial temporal cortex [9–12]. However, in the majority of such studies there is no information on pathology underlying PCA or information on features occurring early in the disease course. Identifying information about underlying pathology is important since different pathologies could have different clinical associations and hence confound the findings in any such study. Additionally, understanding early PCA could have an impact on prognosis and treatment.

In this study, we assessed for the presence of beta-amyloid deposition on Pittsburgh compound B-PET (PiB-PET) scanning in a large cohort of PCA patients and assessed for clinical and FDG-PET findings. Additionally, we assessed for the anatomic correlates of the classic features of PCA and for features that are present early in the disease course.

## Methods

### Patient recruitment

We prospectively recruited all consecutive patients who presented to the Department of Neurology, Mayo Clinic, Rochester, Minnesota, between March 1st, 2013 and August 31st, 2014 with a clinical diagnosis of PCA who also fulfilled our  research criteria for PCA (see below).

Posterior cortical atrophy was defined according to the core criteria previously suggested [4, 13] as follows: (1) insidious onset and gradual progression, (2) presentation of visual complaints in the absence of significant primary ocular disease explaining the symptoms, (3) relative preservation of anterograde memory and insight early in the disorder, (4) disabling visual impairment throughout the disorder, and (5) presence of any of the following: simultanagnosia with or without optic ataxia or oculomotor apraxia, constructional dyspraxia, visual field defect, environmental disorientation or any elements of Gerstmann’s syndrome (acalculia, agraphia, left–right disorientation and finger agnosia).

Patients were included in this study if they also met the following five research criterion for PCA. (1) The chief complaint must be a progressive visuospatial or perceptual problem. (2) The subject must have a normal ophthalmologic examination within 3 months of presentation. (3) Visuospatial/perceptual deficits must be corroborated on neuropsychological testing of spatial/perceptual function. (4) Performance on testing of episodic memory (the Rey Auditory Verbal Learning Test was used for this study) [14] must be superior to (>1.0 *Z* score) performance on testing of visuospatial/perceptual function (the Rey-Osterrieth complex figure test was used) [15]. (5) The neuropsychological test battery must demonstrate that visuospatial/perceptual deficits are more severe than deficits in all other cognitive domains. Patients with symptoms suggestive of severe depression, an infarct or tumor in the occipital, parietal or temporal lobes on head MRI/CT that could have contributed to the presenting syndrome or with medical conditions that could interfere with cognitive performance were excluded.

### Neurological assessments

**Fig. 1:**
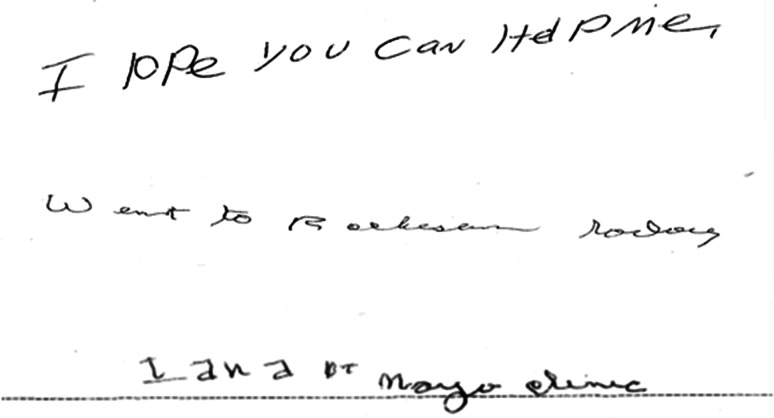
Examples of handwriting from three PCA patients. Note abnormally formed *letters*, abnormal spacing, and variations in *letter* size, consistent with apraxic dysgraphia

All neurological assessments were performed by a behavioral neurologist (KAJ). Demographic variables collected included age, sex, education, handedness, race and disease duration. All patients underwent testing with the Mini-Mental State Examination (MMSE) [16], the Frontal Assessment Battery (FAB) [17], Frontal Behavior Inventory (FBI) [18], apraxia subset of the Western Aphasia Battery (WAB-apraxia) [19], 15-item Boston Naming test (BNT) [20], Movement Disorder Society-sponsored version of the Unified Parkinson’s Disease Rating Scale (parts I, II and III) (MDS-UPDRS) [21], Clinical Dementia Rating (CDR) [22], Neuropsychiatric Inventory (NPI) [23], and a test of famous faces’ recognition [24]. Calculation ability was assessed using the calculation subscore from the Montreal Cognitive Assessment Battery [25]. In addition, the presence/absence of visual hallucinations, rapid eye movement sleep behavior disorder (RBD) and poly-mini-myoclonus at the time of testing were recorded. Rapid eye movement sleep behavior disorder was considered present if the behavior met diagnostic criteria B for rapid eye movement sleep behavior disorder, defined as abnormal, wild flailing movements occurring during sleep (with sleep-related injuries) or movements that are potentially injurious or disruptive [26]. Two of the four patients with RBD had a polysomnography. One showed REM sleep without atonia consistent with RBD. The other had REM sleep with atonia but no non-supine REM sleep was captured. The presence of anomia was assessed using a cut-off of <12 on the BNT. The presence of simultanagnosia was assessed using the Ishihara color plates and the documentation of how many items were recognized on visual inspection of a picture with five overlapping items. Cutoff was below 6/6 for plates and below 5/5 on overlapping pictures based on performance of 10 normal controls that scored 100 % on both tests. Handwriting samples were assessed for evidence of dysgraphia (Fig. 1). The presence/absence of oculomotor apraxia and optic ataxia were assessed on neurological examination. Oculomotor apraxia was defined as the inability to voluntarily direct one’s gaze to a particular point. Optic ataxia was defined as the impairment of goal-directed hand movements towards visually presented targets. Poly-mini-myoclonus was defined as the presence of intermittent and irregular jerks that are visible predominantly in the fingers when the arm is held outstretched and the figures extended, with amplitudes of the jerks just sufficient to produce movements of the joints [27].

### Image acquisition

All patients underwent PiB-PET scanning, while 17 (68 %) underwent FDG-PET, with both acquired using a PET/CT scanner (GE Healthcare, Milwaukee, Wisconsin) as previously described. PET images were acquired after injection of C-11 PiB (average = 596 MBq; range = 292–729 MBq, uptake period = 40 min) and F-18-FDG (average = 540 MBq; range = 366–399 MBq, uptake period = 30 min); both scans were performed as previously described on the same day with 1 h interval between acquisitions [24]. All patients also underwent a standardized MRI imaging protocol at 3.0 T, which included a 3D magnetization prepared rapid acquisition gradient echo (MPRAGE) sequence [24]. A global PiB standardized uptake value ratio (SUVR) was calculated for each PiB-PET scan as previously described [28], with a 1.5 cut-off used for beta-amyloid positivity.

### PET analyses

The FDG-PET images were analyzed both at the individual and group-level. Individual maps of hypometabolism were generated using 3-dimensional stereotactic surface projections. The software package used to perform these analyses was CortexID (GE Healthcare, Waukesha, WI, USA). Automated average *Z* scores generated from Cortex ID were calculated for the following regions: lateral and medial frontal lobes, lateral and medial parietal lobes, temporal lobes, cingulate cortex, occipital lobe, and primary visual cortex. Hypometabolism was considered present if *Z* scores were greater than 2. Additionally, *Z* scores of 2–3 were considered moderate hypometabolism and >3 were considered severe hypometabolism.

Group-level analyses were performed using SPM5 [29]. The FDG-PET scan for each subject was co-registered to the subjects MPRAGE scan using 6 degrees-of-freedom registration. The automated anatomical labelling atlas [30] containing pons was propagated to native MPRAGE space. All voxels in the FDG-PET image were then divided by median uptake of the pons to form FDG uptake ratio images. The MPRAGE scans were then normalized to a customized template, and these normalization parameters were used to also transform the FDG-PET uptake ratio images onto the customized template. Voxel-level comparisons of FDG-PET were performed using two-sided *t* tests in SPM5. The group of PCA subjects was compared to an age and gender-matched group of 20 healthy controls that had undergone identical FDG-PET and MRI acquisitions. These comparisons were performed after correction for multiple comparisons using the false discovery rate (FDR) correction at *p* < 0.001.

Group-level analyses of the PiB-PET scans were also performed using SPM5. As above, the PiB-PET scans were co-registered to the subjects MPRAGE and transformed into customized template space using the normalization parameters from the MPRAGE normalization. All voxels in the PiB-PET images were divided by median uptake in the cerebellum before normalization. Two-sided *t* tests were used to compare the PCA subjects to the 20 healthy controls. These comparisons were performed after correction for multiple comparisons using the false discovery rate (FDR) correction at *p* < 0.001.

### Early PCA

We subdivided the 25 PCA patients based on the median disease duration of the cohort (4 years) in order to assess clinical and FDG-PET features of those that had a short duration of ≤4 years (*n* = 13) and hence could be considered to have early PCA. Disease duration was defined as the time from the onset of the first symptom observed by the patient and the patient’s significant other (both had to agree) to the time of presentation. Ten early PCA patients had an FDG-PET scan performed ≤4 years after onset, available for analysis. Patterns of FDG-PET hypometabolism were compared between the group of early PCA patients and the 20 controls correcting for multiple comparisons using the FDR correction at *p* < 0.001 and between early PCA and the rest of the PCA cohort at *p* < 0.001 uncorrected.

### Statistical analysis

We used JMP software version 10.0.0 (SAS Institute Inc., Cary, NC, USA) to perform statistical analyses. According to convention, statistical significance is represented at two-tailed alpha level of 0.05. Group differences for categorical variables were assessed with the *χ*^2^ test and Fisher’s exact test as appropriate, while differences in continuous variables were assessed using the Kruskal–Wallis test. Spearman Correlation analyses were performed between neuropsychological and clinical symptoms and hypometabolism *Z* scores.

## Results

We identified 25 PCA patients who fulfilled our research criteria for PCA. Two patients were excluded because they presented with aphasia rather than visuoperceptual deficits. The demographic features of all 25 patients are shown in Table 1.

**Table 1:** Demographic features of the PCA cohort

Demographics	Entire PCA cohort (*n* = 25)	Early PCA (*n* = 13)
Males	12 (48 %)	7 (53.9 %)
Right handedness	24 (96 %)	12 (92.3 %)
Age of onset (years)	60 (54–63)	59 (54–62.5)
Age at evaluation (years)	64 (58–70)	61 (56.5–65.5)
Illness duration (years)	4 (3–7)	3 (2.5–4)
Education	16 (13–18)	14 (12–16)
Race (Caucasian)	25 (100 %)	13 (100 %)

Data expressed as *n* (%) or median (IQR)

### Clinical findings

The clinical features of the cohort are shown in Table 2. The most common clinical features in the cohort were simultanagnosia, dysgraphia (Fig. 1), poly-mini-myoclonus and oculomotor apraxia. On neurological testing all scores fell within the normal- mildly affected range except for Ishihara plates in which the average score fell within the severely affected range.

**Table 2:** Clinical and neurological features of the PCA cohort

	Entire PCA cohort (*n* = 25)	Early PCA (*n* = 13)
Clinical features (present/absent)		
Simultanagnosia	23 (92 %)	11 (85 %)
Dysgraphia	17 (68 %)	9 (69.2 %)
Poly-mini-myoclonus	16 (64 %)	9 (69.2 %)
Oculomotor apraxia	13 (56.5 %)	6 (54.6 %)
Anomia	8 (34.8 %)	3 (23.1 %)
Optic ataxia	8 (34.8 %)	0 (0 %)
Visual hallucinations	5 (20 %)	3 (23.1 %)
REM sleep behavior disorder	4 (16 %)	3 (23.1 %)
Neurological test scores		
MMSE (/30)	25 (20–28)	26 (24–29)
FAB (/18)	14 (11–16)	15 (12–15.5)
FBI (/72)	12 (8–18.5)	11 (5.5–17.5)
WAB apraxia (/60)	55 (52–59)	56 (53–58.5)
MDS-UPDRS I (/52)	7 (5.5–10.5)	7 (5.5–9.5)
MDS-UPDRS II (/52)	6 (1.5–1.3)	4 (1–7)
MDS-UPDRS III (/132)	2 (0.5–4)	2 (0.5–3)
CDR sum of boxes (/18)	3.5 (2–6.8)	2.5 (1.8–4)
NPI total score (/36)	4 (2–6)	4 (2.5–5.5)
Boston naming test (/15)	13 (10–14)	13 (11–15)
Famous face recognition (/10)	9 (7–10)	10 (9–10)
Calculations (/5)	2 (0–3)	2 (0.5–3)
Ishihara (6 plates)	0 (0–2)	1.5 (0–3)

Data as (%) or median (IQR)

### Single subject FDG-PET analysis

The *Z* scores for hypometabolism and the number of affected patients are shown in Table 3. Severe hypometabolism was seen in the right parietal lobe in 16 (94.1 %) patients, left parietal lobe in 14 (82.4 %) extending through the right and left medial parietal lobes in 16 (94.1 %) and 14 (82.4 %) patients and finally involving the right and left occipital lobes in 14 (82.4 %) and 15 (88.2 %) patients. Mild hypometabolism was present in bilateral frontal and temporal lobes, extending through the left and right posterior cingulum, and the bilateral visual cortex. Both medial frontal cortices and anterior cingulum remained unaffected in all the patients of our cohort.

**Table 3:** *Z* scores of hypometabolic patterns in PCA

	*Z* scores (median)	No (%) patients with *Z* score >2
**Parietal lobe, R**	**4.26**	**16 (94.1)**
**Parietal lobe, L**	**3.75**	**14 (82.4)**
**Medial parietal, R**	**3.36**	**16 (94.1)**
**Occipital lobe, R**	**3.18**	**14 (82.4)**
*Occipital lobe, L*	*2.97*	*15 (88.2)*
*Medial parietal, L*	*2.86*	*14 (82.4)*
*Temporal lobe, L*	*2.61*	*11 (64.7)*
*Temporal lobe, R*	*2.48*	*13 (76.5)*
Posterior cingulate, L	1.87	6 (35.3)
Posterior cingulate, R	1.80	8 (47.1)
Frontal lobe, L	1.79	6 (35.3)
Visual cortex, R	1.64	6 (35.3)
Visual cortex, L	1.49	5 (29.4)
Frontal lobe, R	1.38	6 (35.3)
Medial frontal, L	1.16	0 (0)
Anterior cingulate, R	0.98	0 (0)
Medial frontal, R	0.97	0 (0)
Anterior cingulate, L	0.76	0 (0)

Values are ordered from most affected to least affected. Bold and italicized values represent degree of severity (>3.00 = severe, 2.00–3.00 = moderate, 1–1.99 = mild)

### Group analysis of FDG-PET using SPM

**Fig. 2:**
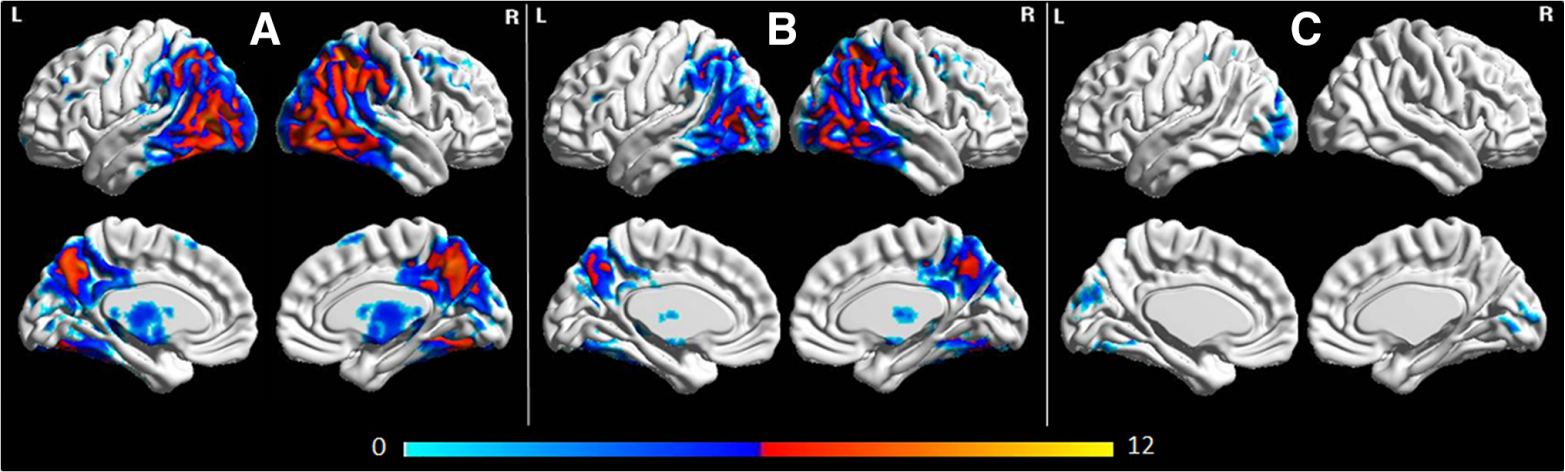
Three-dimensional brain renderings showing FDG-PET hypometabolism in the entire PCA cohort compared to controls (**a**), the early PCA subjects compared to controls (**b**), and early PCA compared to the rest of the PCA patients (**c**). Renders were generated using the BrainNet Viewer (http://www.nitrc.org/projects/bnv/)

The regions of hypometabolism observed in all 17 patients are shown in Fig. 2. There was reduced metabolism bilaterally throughout lateral parietal and occipital cortices, precuneus and posterior cingulate, lateral posterior temporal cortex and medial occipital cortex, particularly in the right hemisphere, compared to controls.

### Associations between classic PCA clinical features and FDG-PET

Simultanagnosia was associated with hypometabolism in the right occipital lobe (*p* = 0.0054), right posterior cingulum (*p* = 0.0186) and the right visual cortex (*p* = 0.0193). In contrast, optic ataxia was associated with hypometabolism in left occipital lobe (*p* = 0.0109) and left visual cortex (*p* = 0.0339) and oculomotor apraxia with left parietal lobe (*p* = 0.0357), left posterior cingulate gyrus (*p* = 0.0157) and left medial parietal cortex (*p* = 0.0175). There was no association between dysgraphia and acalculia and the hypometabolism patterns.

### Early PCA

Demographic features were similar between early PCA and all PCA patients (Table 1). Only four features were present in more than 50 % of the early PCA patients including simultanagnosia, dysgraphia, poly-mini-myoclonus and oculomotor apraxia (Table 2). Only simultanagnosia was present in 85 % of the early PCA patients. The only feature than differed by more than 20 % between early PCA and the entire cohort was optic ataxia. Three patients had disease duration of less than 2 years of which two had simultanagnosia and poly-mini-myoclonus. Performance on neurological testing in early PCA was similar to performance in the entire PCA cohort although the early PCA patients were in general less cognitively impaired.

In the SPM analysis (Fig. 2), the early PCA group showed a similar pattern of hypometabolism to the entire cohort although there was less involvement of the occipital cortex in early PCA compared to the rest of the cohort.

### PiB-PET analysis

**Fig. 3:**
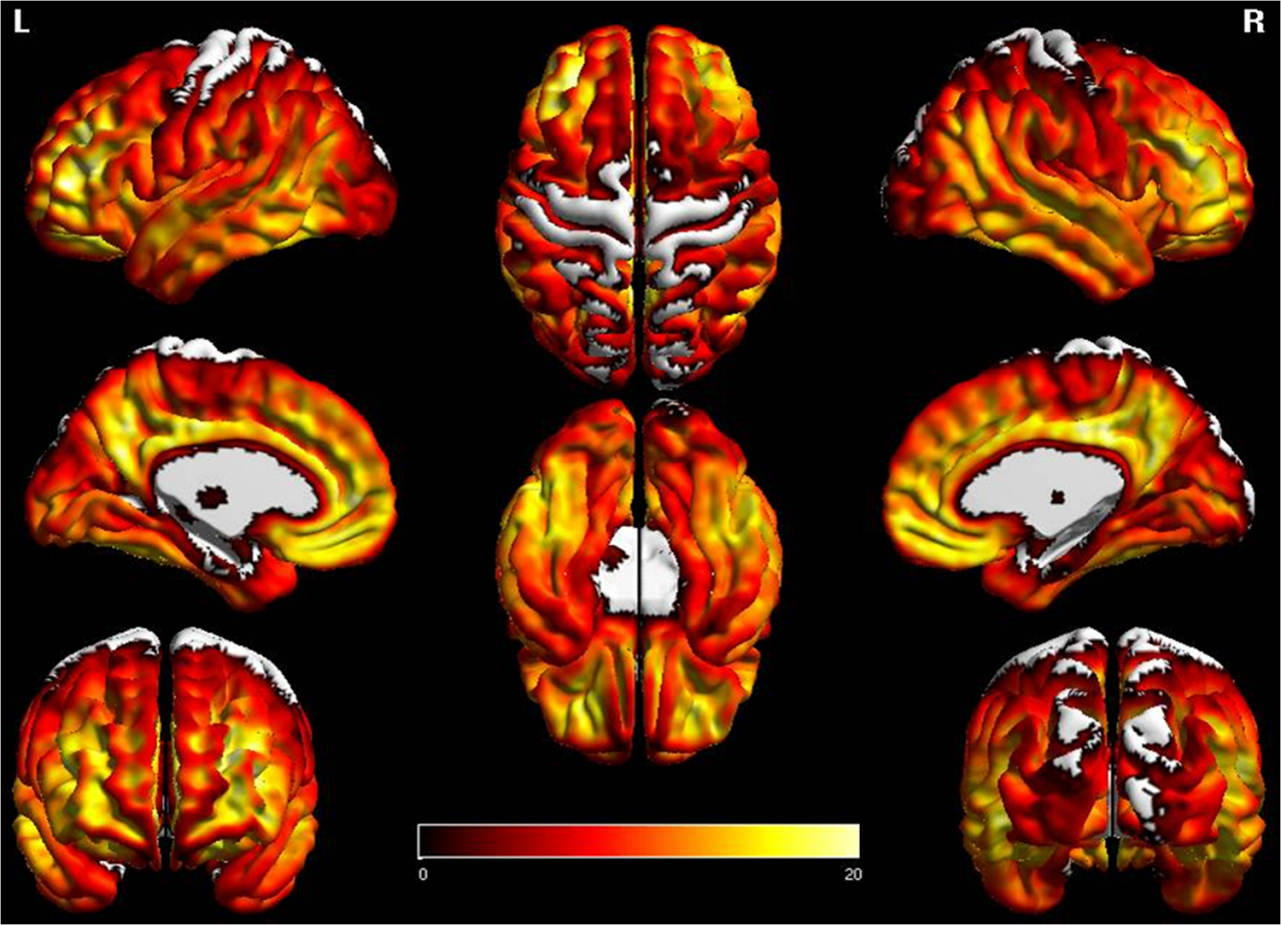
Three-dimensional brain renderings showing PiB-PET uptake in the entire PCA cohort compared to controls. Renders were generated using the BrainNet Viewer (http://www.nitrc.org/projects/bnv/)

All 25 PCA patients had an SUVR ratio >1.50 and hence were amyloid positive. The average SUVR ratio for all 25 PCA patients was >2.19 (±0.35). Twenty of the 25 (90 %) PCA patients had an SUVR >2.00. The regional pattern of PiB-PET uptake is shown in Fig. 3. Widespread PiB-PET uptake was observed with most severe uptake in lateral and medial prefrontal, lateral temporal and medial parietal cortex.

The average SUVR ratio of the early PCA patients was 2.08 (±0.31). Eleven of the 13 (87 %) early PCA patients had an SUVR ratio >2.00. There was no difference in SUVR ratio between early PCA and the rest of the PCA cohort.

## Discussion

This is one of the largest prospective studies to date of a cohort of patients with a clinical diagnosis of PCA that underwent FDG-PET and amyloid PET imaging. The study identifies important clinical, cognitive and neuroimaging features of PCA.

While the clinical features of our PCA patients are unsurprisingly similar to previously reported PCA cohorts [2, 4, 13, 31–33], our study design allowed us to further refine the clinical syndrome of PCA. Our PCA cohort had an average age of onset of 60 years which might suggest that PCA should be considered an early onset dementia. However, we did not have any subjects presenting less than 45 years and hence PCA would not be considered a young onset dementia, based on our previous definitions [34]. Simultanagnosia was noted to be the most common clinical feature in our cohort in general, and was also the most common feature of early PCA, affecting 85 % of patients with disease duration of 4 years or less. Dysgraphia and oculomotor apraxia were also common features of PCA, and of early PCA. Poly-mini-myoclonus however, is not a feature that is considered, or typically assessed, in patients with PCA yet it was more common than some of the features typically associated with PCA, such as oculomotor apraxia and optic ataxia. We previously reported an association between poly-mini-myoclonus and PCA [35], although our previous study was retrospective in nature and clearly underestimated the frequency of this clinical feature compared to the current prospectively recruited cohort, which shows that poly-mini-myoclonus is a common feature of PCA. In contrast to simultanagnosia, oculomotor apraxia and poly-mini-myoclonus, our data suggested that optic ataxia, while relatively common in PCA, is a later feature of PCA. In fact, optic ataxia was not identified in early PCA. Anomia was also observed in our PCA cohort, as previously reported [4, 33, 35, 36], and is most likely due to involvement of the left temporal lobe. We also found that approximately 20 % of our cohort had visual hallucinations or RBD, with the frequency similar in early PCA and across the entire cohort. This suggests that hallucinations and RBD when associated with PCA are both likely to be observed early in the disease course. These two features are not frequently reported in PCA, but are suggestive of underlying Lewy body disease [7, 35], given these are classic features of dementia with Lewy bodies [37]. Acalculia, a feature of the Gerstmann’s syndrome, was striking and was also observed in early PCA. Prosopagnosia was noted to be present in some of the patients but was relatively mild when present in most instances and was most likely perceptual, as opposed to agnostic in nature. Neuropsychiatric, behavioral, executive and extrapyramidal features were noted to be absent to mild in PCA. Ideomotor apraxia was not a common feature and when present also appeared to be mild in most patients.

On FDG-PET analysis, we observed marked hypometabolism of the bilateral parietal and occipital lobes, showing involvement of these regions in more than 80 % of the PCA patients. Mild hypometabolism in bilateral frontal lobes, posterior cingulate and the bilateral visual cortex was observed in 50 % of the PCA patients. The bilateral anterior cingulate and medial frontal cortex appears to remain unaffected. These findings concur with previous PET studies [9–12]. All 25 PCA patients were amyloid positive with very high SUVR ratios. This suggest, with high certainty that the underlying pathology in all of our PCA patients is Alzheimer’s disease. It is also possible however, although less likely, that in some of our cases amyloid deposition is co-occurring with another pathology, such as corticobasal degeneration that has been reported in a couple of PCA patients [6]. It is also unlikely that our patients suffered from Creutzfeldt-Jakob disease, given the disease durations and the fact that amyloid imaging is negative in Creutzfeldt-Jakob disease [38]. Furthermore, large amplitude myoclonic jerks, a feature of Creutzfeldt-Jakob disease, was not observed in our cohort. Our PCA patients instead showed poly-mini-myoclonus. Therefore, our cohort is likely very homogeneous, pathologically, although it is possible that in some patients there is co-existing Lewy body pathology.

Our association analyses allowed us to investigate the hypometabolic associations of the clinical features observed in PCA. Simultanagnosia was associated with hypometabolism in the right occipital lobe, posterior cingulum and visual cortex. These findings are expected as simultanagnosia generally reflects the severity of damage in the visual associative cortex without the involvement of the lateral parietal cortex [2, 39]. However, in our study it was found to be associated only with right-sided involvement. Optic ataxia was associated with involvement of the left occipital and visual cortex, while oculomotor apraxia with left parietal lobe and posterior cingulum. These results further quantify the neural correlations of oculomotor apraxia to the posterior parietal cortices. In fact, an fMRI study recently reported finding an association between the lateral occipitoparietal junction (dorsal stream) and changes in graspable stimuli and visually guided reaching to grasp [40, 41]. The optic ataxia findings suggest that occipital lobe involvement needs to be present before this PCA feature will emerge. We did not find any association between two of the features of Gerstmann’s syndrome (acalculia and agraphia) with any hypometabolic patterns, which could be due a small sample size of our cohort; however, these findings have been shown to be associated with the left parietal cortex by another group [42].

One of the important aspects of our study was the assessment of clinical and FDG-PET features of early PCA. We observed little difference in early PCA compared to the entire cohort suggesting most features of PCA will be present early in the disease course. The only exception appeared to be optic ataxia which was not a feature of early PCA. Interestingly, the presence of optic ataxia was associated with left occipital lobe involvement which was a region found to be less affected in early PCA. And although we do not have longitudinal data, we can speculate that progressive involvement likely spreads from the right parietal lobe across to the left occipital lobe, which would explain the later emergence of optic ataxia.

All 25 PCA patients had SUVR ratios consistent with beta-amyloid deposition. Beta-amyloid is one of the two major proteins required for a pathological diagnosis of Alzheimer’s disease; tau being the other. Our group analysis of PiB did not show a regional pattern of beta-amyloid deposition unique to PCA. Prefrontal, temporal and medial parietal involvement has also been observed with other Alzheimer’s disease related syndromes [28, 43, 44]. The lack of beta-amyloid deposition being more striking in the occipitoparietal regions, mirroring the pattern of hypometabolism on FDG-PET, is consistent with pathological studies demonstrating greater tau, but not beta-amyloid deposition in these regions [4]. The subjects in this study all met clinical criteria for PCA [4, 13]. All subjects also met stringent research criteria for PCA. Given that we did not have any amyloid negative subjects in our cohort, it appears that our research criteria may be more specific to underlying Alzheimer’s disease than the clinical criteria, since other PCA cohorts have found subjects with non-Alzheimer’s pathologies [5, 6]. We have previously reported two subjects who presented with aphasia but were agnostic to their visual deficits [45]. Visuospatial/perceptual deficits were strikingly abnormal on neuropsychological testings and was the most severely impairment cognitive domain in both. Such subjects remain controversial in terms of classification. In fact, both were excluded from this study as they did not meet our research criteria for PCA.

The major strength of this study is the large number of PCA cases that were prospectively recruited and underwent amyloid and FDG-PET imaging. Another strength was the assessment of PCA subjects with short disease duration allowing the assessment of early features of PCA. A limitation of the study was that not all subjects underwent FDG-PET. In addition, pathological confirmation of Alzheimer’s disease was not available. The lack of autopsy also meant we could not confirm an association between the presence of hallucinations and RBD and co-existence of Lewy body disease.
